# Nanoporous smartPearls for dermal application – Identification of optimal silica types and a scalable production process as prerequisites for marketed products

**DOI:** 10.3762/bjnano.10.162

**Published:** 2019-08-08

**Authors:** David Hespeler, Sanaa El Nomeiri, Jonas Kaltenbach, Rainer H Müller

**Affiliations:** 1 Pharmaceutical Technology, Institute of Pharmacy, Freie Universität Berlin, Kelchstraße 31, 12169 Berlin, Germany; 2 Department for Mathematics, Physics and Chemistry, Beuth Hochschule für Technik Berlin, Luxemburger Straße 10, 13353 Berlin, Germany; 3 Institute of Functional Interfaces, Karlsruher Institute of Technology, Hermann-von-Helmholtz-Platz 1, 76344 Eggenstein-Leopoldshafen, Germany

**Keywords:** amorphous dispersion, bioavailability enhancement, dermal delivery system, rutin, smartPearls, solubility enhancement

## Abstract

smartPearls are a dermal delivery system for poorly soluble active agents, consisting of nanoporous silica particles loaded with a long-term stable, amorphous active agent in its mesopores (2–50 nm). The amorphous state of the active agent is known to increase dermal bioavailability. For use in marketed products, optimal silica types were identified from commercially available, regulatory accepted silica. In addition, a scalable production process was demonstrated. The loading of the particles was performed by applying the immersion–evaporation method. The antioxidant rutin was used as a model active agent and ethanol was applied as the solvent. Various silica particles (Syloid^®^, Davisil^®^) differing in particle size (7–50 µm), pore diameter (3–25 nm) and pore volume (0.4–1.75 mL/g) were investigated regarding their ease of processing. The evaporation from the silica–ethanol suspensions was performed in a rotary evaporator. The finest powders were obtained with larger-sized silica. The maximum loading staying amorphous was achieved between 10% and 25% (w/w), depending on the silica type. A loading mechanism was also proposed. The most suitable processing occurred with the large-sized Syloid^®^ XDP 3050 silica with a 50 µm particle size and a pore diameter of 25 nm, resulting in 18% (w/w) maximum loading. Based on a 10% (w/w) loading and the amorphous solubility of the active agent, for a 100 kg dermal formulation, about 500 g of loaded particles were required. This corresponds to production of 5 kg of loaded smartPearls for a formulation batch size of a ton. The production of 5 kg (i.e., about 25 L of solvent removal) can be industrially realized in a commercial 50 L rotary evaporator.

## Introduction

Many interesting active agents in pharma and cosmetics are poorly soluble. Active agents that are poorly soluble in water but soluble in lipophilic media can easily be formulated as creams or gels (e.g., the popular coenzyme Q10). The problems start when the active agents are poorly soluble both in aqueous and lipophilic/organic media. Classical examples are antioxidants (e.g., rutin, hesperidin), which are presently en vogue in cosmetics for antipollution products (e.g., the “molecular barrier” against reactive oxygen species (ROS), infrared (IR) radiation and blue light from computers) [1–2]. For the delivery of such molecules, efficient delivery systems are the only solution, because the application of simple suspensions to the skin normally does not provide a sufficient dermal bioavailability.

Classic delivery systems such as liposomes [3] or solid lipid nanoparticles (SLNs) [4–5] do not work because the active agents do not dissolve in the lipidic phase of these systems. A simple but very effective approach is to increase the saturation solubility of these active agents. This leads to an increased concentration gradient between the formulation and skin, *C*_s_–*C*_skin_, and thus to an increased diffusional flux into the skin. Moreover, using complexes with polymers or cyclodextrins, for example, can be of limited effect because of insufficient release of the molecules from such complexes (i.e., too high binding constants) [6–7]. Additionally, many molecules are not able to form such complexes. A highly effective solution is the dermal administration of nanocrystals (trade name smartCrystals^®^) [8–10]. These materials have been on the market as commercial dermal cosmetic products since 2007 (e.g., hesperidin, la prairie Switzerland) [11–12]. They can be considered as the current “gold standard”.

The trick with nanocrystals is that the physicochemical properties on the micrometer scale differ from those on the nanometer scale, and this results in distinct changes (e.g., the saturation solubility distinctly increases) [13]. In general, amorphous materials have an even higher *C*_s_ than nanocrystalline materials [14]. Thus, it would be more effective to use active agents in the amorphous state. However, the amorphous state is physically unstable. Because of their high free energy, amorphous phase materials tend to recrystallize [15], especially in the presence of liquids [16]. This has hindered the broad application of amorphous active agents in dermal formulations. The company Capsulution (Berlin, Germany) incorporated active agents in the amorphous state inside the pores of silica particles with mesopores (2–50 nm) [17] using the technology from CapsMorph for oral administration [18]. With this, the amorphous state could be stabilized over the course of years [19]. Porous silica particles are commercially available, for example, from Grace, Merck Millipore and Formac [20]. They are considered nanoporous materials because their pore diameter is on the nanometer scale [21]. The silica they used had so-called "mesopores", i.e., pores with dimensions in the range of 2–50 nm. In 2006 this delivery technology was transferred from the oral to the dermal administration route [22] by applying a technology called smartPearls [23]. The name was changed to smartPearls to clearly differentiate them from silica used for oral administration.

smartCrystals are found in products on the cosmetic market, because industrial large-scale production is possible and an industrial supplier is available (Dr. Rimpler GmbH, Germany) for manufacturers of cosmetics. smartCrystals are crystals of nanometer dimension, typically 200–400 nm, which can be produced on a large scale by bead milling or high-pressure homogenization. Skin penetration studies showed that the smartPearls were actually superior to the nanocrystals [8,24–25]. However, the market introduction in final cosmetic products was blocked due to the lack of an industrial supplier of active agent-loaded smartPearls. To establish an industrial supply, an industrially feasible production method is required, which is the focus of this work.

Silica particles can be loaded by co-milling [26–27], however, with this technique, a large portion of the active agents are not incorporated into the pores. Loading can be performed by supercritical carbon dioxide processing [28], but it is expensive. Loading is also possible by the impregnation–evaporation method [29], but this is less suitable for large-scale production. In this study, the immersion–evaporation method [30] was applied and systematically investigated to define large-scale production parameters for an industry friendly one-step production process. Rutin was used as the model active agent because it has high application potential in cosmetic products as well as in dermal pharmaceutical products [31]. A loading mechanism for this industrial process is proposed in this work. In addition, the concentration of smartPearls in the final dermal products are estimated based on the achieved loadings and on the solubility data. This work could serve as a guideline for manufacturers of dermal products. Different types of silica were investigated having various parameters (e.g., particle size, pore diameter, pore volume) in order to identify particles which are most easy to process.

## Materials and Methods

### Materials

Rutin with a purity of 95% was purchased from Denk Ingredients (Munich, Germany). Various mesoporous silica particles (Table 1) with pore diameter of 3 nm (Syloid^®^ AL-1 FP), 6 nm (Davisil^®^ LC 60 Å 12 µm), 10 nm (Syloid^®^ 72 FP), 17 nm (Syloid^®^ 244 FP), and 25 nm (Syloid^®^ XDP 3050) were kindly provided by Grace GmbH & Co. KG (Worms, Germany). Ethanol, isopropanol, butanol, acetone, ethyl acetate, acetonitrile and dimethyl sulfoxide (DMSO) in gradient grade were purchased from VWR (Darmstadt, Germany), and purified water produced by a Milli-Q system from Merck Millipore (Darmstadt, Germany) was used.

**Table 1:** Silica particles used in this work and their properties according the certificate of analysis (manufacturer: W. R. Grace & Co., USA).

Product code	Pore diameter [nm]	Specific surface area [m^2^/g]	Pore volume [mL/g]	Particle size [µm]

Syloid^®^ XDP 3050	25	300	1.8	50
Syloid^®^ 244 FP	17	380	1.6	3
Syloid^®^ 72 FP	10	370	1.2	5
Davisil^®^ SP53D	6	550	0.9	12
Syloid^®^ AL-1 FP	3	740	0.4	7

### Methods

#### Solubility investigation of rutin

To assess the maximum solubility of rutin, rutin suspensions were prepared with different solvents (dimethyl sulfoxide (DMSO), ethanol, isopropanol, butanol, acetone, ethyl acetate, acetonitrile, and water) (*n* = 1). The suspensions with 20% (w/w) rutin were shaken overnight at room temperature, centrifuged, filtered and the rutin concentration in the filtrate was analyzed after dilution in ethanol (by a factor of 100–1000) by UV spectrophotometry (UV-1700 PharmaSpec, Shimadzu, China) at 360 nm. The evaluation was conducted by the provided “UVprobe” software (version 2.21). The concentration was calculated based on the calibration curves determined in ethanol.

#### Particle loading

Prior to loading, the silica particles were dried in an oven at 120 °C for at least 2 h. The saturated rutin solution was produced by preparing a 2% (w/w) rutin suspension in ethanol (96 vol %), heating it under agitation to 60 °C for 1 h and subsequent filtration. After cooling, it was checked that no rutin crystals precipitated out. The rutin content was determined prior using the rutin solution for loading. For loading, 3 g of silica particles were dispersed in a respective volume of rutin ethanol solution. The volume used depended on how much rutin should be loaded into the silica particles (increasing amounts of solution with increased percent of loading). The suspension was stirred for 5 min to achieve a fine dispersion of the particles. Then the suspension was placed into a rotary evaporator (Büchi, Germany). The solvent evaporation took place at 40 ± 2 °C and 150 ± 10 mbar, until a film was formed on the wall of the evaporation flask. The evaporation time depends on the amount of solvent used (i.e., increased evaporation time with an increase of loading percentage). For removal/minimization of solvent residues, a secondary drying phase at 40 ± 2 °C and 20 mbar was performed for 30 min. All fractions presented are weight fractions unless otherwise stated. Each loading for each type of silica was performed once.

#### Differential scanning calorimetry (DSC)

The physical state of the silica particles, the rutin raw active agent powder and the rutin smartPearls was investigated by differential scanning calorimetry (DSC, Mettler Toledo GmbH, Germany) and calculated by the provided STARe software (version 12.10b). Exact amounts between 1–5 mg of rutin-loaded silica based on the respective mass of rutin (with a target of 0.5–1 mg) were weighed in a punctured 40 µL aluminum pan and sealed (*n* = 1). The measurements were performed at a heating rate of 20 K/min between 25 and 300 °C under 80 mL/min nitrogen purge.

#### X-ray diffraction (XRD)

To determine the amorphous state and possible residual crystal fractions of rutin in smartPearls, XRD was performed using a Bruker D8 (Bruker, USA) instrument (*n* = 1). A scan rate of 0.02°/s (2θ = 2–60°) was applied and the goniometer was equipped with a Cu anode (Cu Kα, λ= 0.15406 nm) at a voltage of 40 kV and current of 35 mA.

#### Light microscopy (LM) and scanning electron microscopy (SEM)

Light microscope imaging was performed using a Motic microscope (BA210, Motic Deutschland GmbH, Germany) with a Moticam camera and the software Motic Images Plus at 100, 400, and 1,000-fold magnification. SEM was performed at 10,000-fold magnification using a Zeiss DSM950 (Carl Zeiss AG, Germany) instrument. The samples were sputter-coated with gold–palladium in an argon atmosphere at 15–20 kV at 0.05 mbar for four minutes.

#### Determination of recovery rate

For photometric analysis, a precisely weighed amount of rutin-loaded silica particles (around 25 mg) was dispersed in 5 mL of DMSO. DMSO was used because of the high rutin solubility in this solvent. After 10 min of shaking and 10 min of ultrasonication the suspension was centrifuged at 10,000 rpm for 10 min and an aliquot of the supernatant was diluted in ethanol by a factor of 100. Absorption spectroscopy was performed at 360 nm on a PharmaSpec UV-1700 instrument (Shimadzu, China). The peaks were evaluated using the PharmSpec software “UVprobe” (version 2.21).

High-performance liquid chromatography (HPLC) measurements of the same samples were performed at 25 °C using a Kontron 400 system (Kontron Instruments GmbH, Germany) equipped with a 20 µL loop and a Eurosper 100-5 C18 column (250 × 4 mm, 5 µm particle size) column. Determination was performed at 360 nm with 20:80 acetonitrile/acetate buffer (pH 4.8, 0.1 mol/L) as a mobile phase and at a flow rate of 1.25 mL/min. The peaks were evaluated with the provided software, KromaSystem 2000 (version 1.82).

#### Solubility determination of rutin dissolved from smartPearls

The solubility of the raw rutin active agent powder and the rutin smartPearls was monitored over one hour in 0.15 molar NaCl solution using in situ UV–vis measurements (Sirius inForm^®^, UK) with a fiber optic collector and a path length of 5 mm. The 0.15 molar NaCl solution was placed in a beaker and the aqueous phase was tempered to 25 °C. After calibration of the pH electrode and the UV–vis fiber optic, the sample was manually added immediately after and the UV–vis measurement with the fiber optic was started. The spectra were corrected using the Tyndall Rayleigh correction function. The different concentrations of raw active agent powder and rutin smartPearls were investigated, limited by a maximum overall absorption up to 2.5. The pH was determined over the whole measurement period.

## Results and Discussion

### Considerations for selection of the production method/variables

The production method should yield a high loading in the pores and complete transformation of the active agent into the amorphous state, thus co-grinding was excluded as a potential production method. Also excluded was supercritical CO_2_ processing, due to production cost reasons, complexity of the process and cost of a production unit. Ideally, the processing method should be a one-step method. This excludes the impregnation–evaporation process, because in many cases one impregnation step is not sufficient to reach the anticipated loading. Thus, the immersion–evaporation process was selected.

**Figure 1:**
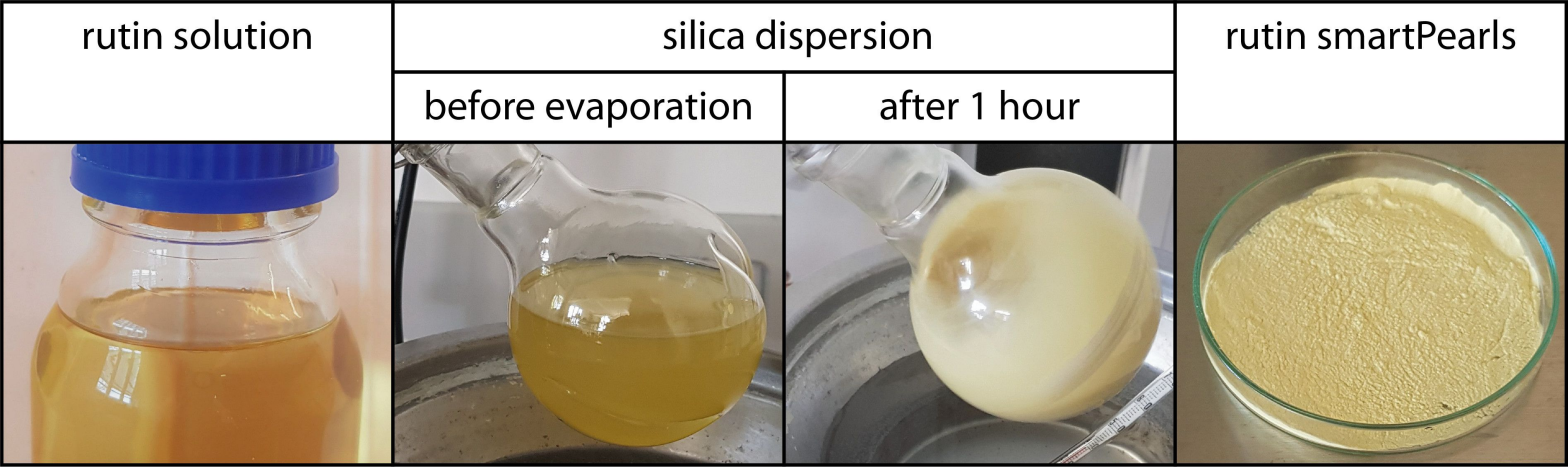
From left to right: rutin/ethanol solution, silica dispersed in rutin solution attached to the rotary evaporator, result after the first hour of solvent evaporation, and the obtained rutin-loaded silica powder, SP53D (right).

The evaporation time of the solvent removal step ranged from 75 to 180 min, depending on the solvent volume. After the second drying step, the obtained free flowing, dry powder was removed. When a film was formed on the glass wall, the film was removed from the wall using a spatula. Potential aggregates were deaggregated by stirring the powder, whereby the aggregates were easily dispersed. The results from the processing steps are shown in Figure 1.

The principle of the procedure was to suspend the particles in an amount of solvent containing the total amount of active agent to be loaded into the pores of the particles. During the evaporation process, the concentration of the active agent increases, precipitation takes place, and continues until complete removal of the solvent. It was expected that precipitation takes place preferentially in the pores because of the largest available surface area. Minor precipitation on the outer shell of the particles as a thin layer represents no problem as long as the active agent on the surface remains amorphous. It is obvious that in addition to the inner surface in the pores, localization on the surface of silica particles also takes place. It is important to avoid the formation of crystals of the active agent on the shell of the particles. This was checked by light microscopy and scanning electron microscopy.

To study the suitability of the process, silica particles differing in particle size, pore diameter and pore volume were selected (Table 1). The investigation should also provide evidence as to which particle type is optimal to achieve maximum loading.

### Solubility of rutin in solvents

A compromise had to be made between the highest possible solubility of rutin and the tolerability of the solvent by the skin. Furthermore, processing parameters such as evaporation temperature were crucial. DMSO was used in previous studies with the impregnation–evaporation method [24] because it has a high solubility and thus reduces the number of loading steps. The disadvantage was the long time required for solvent removal at high temperatures (>80 °C), which can cause degradation of the active agent. In addition, it is tedious to remove DMSO efficiently by rotary evaporation. Although DMSO can be found in products applied to the skin, it is less skin friendly. The efficient removal of critical solvents below the ppm specification is also a cost factor in the production process. Thus, a different, more skin-tolerable solvent was desirable.

Table 2 shows the obtained solubility of rutin in the various tested solvents. Rutin has the highest solubility in DMSO (17%), as expected, but it was decided to select ethanol as a compromise. With a maximum of 2%, the rutin solubility is substantial lower in ethanol than in DMSO, but distinctly higher than in acetone (0.8%) and the other organic solvents. The rutin/ethanol solution had a clear, yellowish appearance.

**Table 2:** Solubility of rutin at room temperature in various solvents (w/w %) and the macroscopic appearance of the solutions.

Solvent	Solubility [% (w/w)]	Appearance

DMSO	17.05	brown
ethanol	2.09	clear, bright yellow
*n*-butanol	2.07	clear, bright yellow
isopropanol	1.85	clear, bright yellow
acetone	0.83	pale yellow
ethyl acetate	0.42	mildly cloudy
acetonitrile	0.22	mildly cloudy
water	0.03	cloudy, slight yellow

### Loading of silica particles

**Figure 2:**
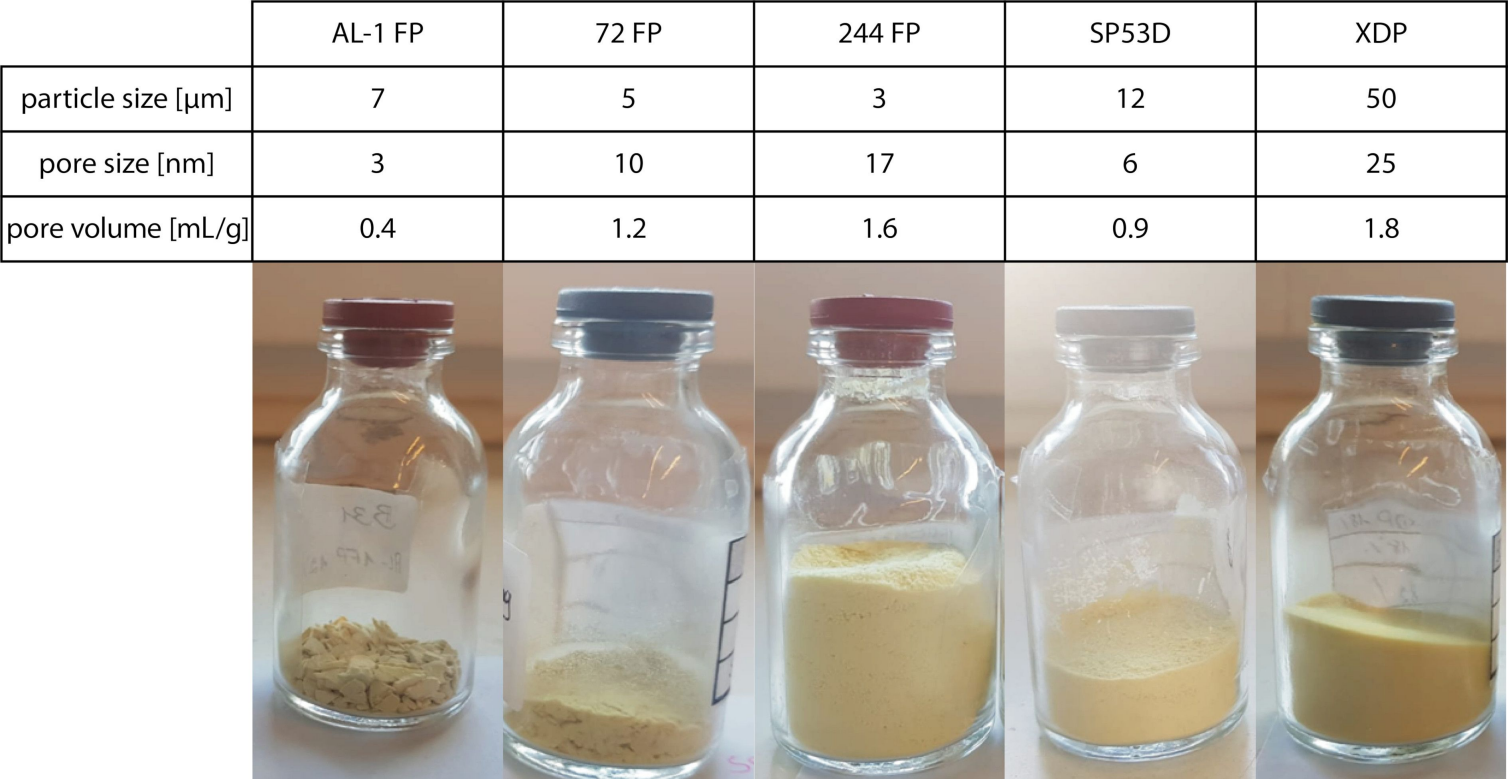
Macroscopic appearance of rutin-loaded silica powders, with decreasing agglomeration tendency from left to right (see text for details).

Different silica materials (Table 1) were loaded with rutin by dispersing them in a saturated rutin/ethanol solution and evaporating the solvent in a rotary evaporator, resulting in dry powders. Nitrogen adsorption/desorption studies were performed, showing little decrease in the pore diameter, but a reduction of the pore volume, supporting the bottom-to-top filling hypothesis of the pores [32–33]. Depending on the particle size and the pore diameter, the powders showed different macroscopic appearances (Figure 2). The silica AL-1 FP tended to form aggregates, and possessed particle diameter of 7 µm. The silica materials with the smallest pore diameter, 244 FP and 72 FP (particle diameter 3 and 5 µm; pore diameter 10 and 17 nm, respectively), showed slight agglomeration. SP53D, having a medium-sized particle size of 12 µm, showed almost no agglomeration, and at a higher loading of 28% and 30%, a film formed on the wall of the evaporator glass. The XDP 3050 particles, having the largest diameter of 50 µm (pore diameter of 25 nm), showed neither agglomeration nor film formation.

It is known that silica particles become more adhesive with decreasing size, which is easily explainable by powder technology. With decreasing particle size, the surface and contact area increase, thus promoting particle–particle interaction. Additionally, with increasing pore diameter, the agglomeration tendency decreases. The particles with a strong agglomeration tendency (AL-1 FP) have the smallest pore diameter of 3 nm, and additionally, the smallest pore volume. It is assumed that the pore parameters only play a significant role in agglomeration tendency when the pore volume is completely filled with active agent or when the pores are too small; this implies that an overloading takes place and the precipitating active agent acts as a kind of glue between the silica particles. This was assumed for the AL-1 FP samples since rutin with a molar weight of 610.5 g/mol and a minimum projection area of 9.2 nm^2^ was assumed to be too large to efficiently diffuse into the pores prior to precipitation. Thus, such small pores promote agglomeration on the outside of the pores. Based on the behavior observed, silica particles with a larger size, larger pores and larger pore volume are easier to process on a large scale.

### Determination of amorphous-phase content

The degree of amorphous phase of the loading was determined by performing DSC and XRD. An amount of 5% rutin in the physical mixture with amorphous unloaded silica was detectable by DSC. The analysis of the physical mixture of silica with crystalline rutin powder showed that an amount of only 3% crystalline rutin was clearly detectable by XRD, while 1.5% was hardly detectable (XRD not shown).

**Figure 3:**
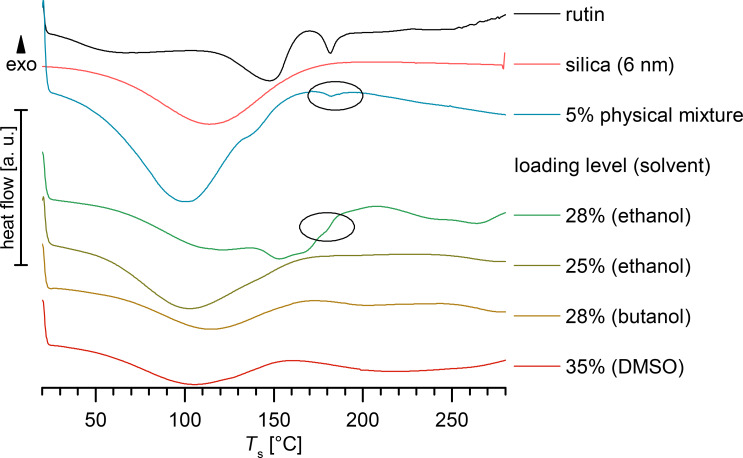
DSC thermograms of crystalline rutin, unloaded silica, a physical mixture of 5% rutin, to be compared to rutin-loaded SP53D processed with ethanol (28% and 25%), butanol (28%) and DMSO (35%).

Figure 3 shows the DSC thermograms for the maximum-loaded silica, SP53D, which was loaded using different solvents and compared to unloaded silica, rutin and their physical mixture. In the physical mixture, 5% rutin was clearly detectable. No rutin peak was detectable for loadings up to 25% with ethanol, 28% with butanol and 35% with DMSO. Crystalline rutin was clearly detectable for a 28% loading with ethanol (detectable in temperature range 175–190 °C). It was thus concluded that the limit of loading in the amorphous state was 25% using ethanol as the solvent. As reported by Jin [24], loadings of 32% are achievable with DMSO using the impregnation–evaporation method. The same loading, or even higher, can be achieved using the immersion–evaporation method. However, the immersion–evaporation method is scalable and thus of more practical relevance. Since DMSO is a difficult to process solvent and more expensive than ethanol, a loading of 25% using ethanol is considered favorable.

**Figure 4:**
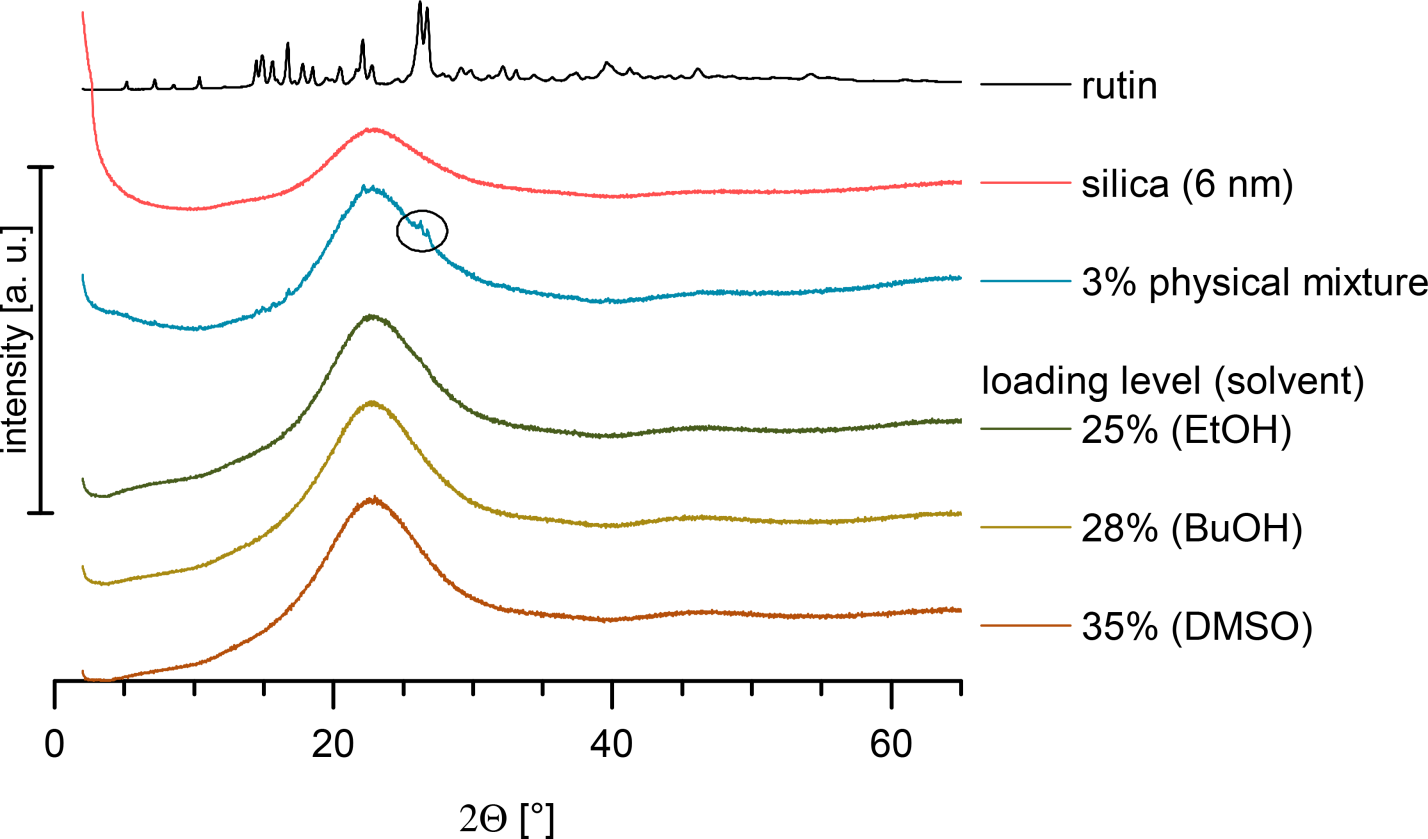
XRD of rutin, unloaded silica (SP53D), and a physical mixture (3% rutin) in comparison to the maximum loading with rutin-loaded silica SP53D using different solvents (ethanol, butanol, DMSO).

Figure 4 shows XRD patterns of rutin in comparison to unloaded amorphous silica, crystalline rutin and their physical mixture. The respective XRD patterns of rutin-loaded SP53D silica with ethanol (25% loading), butanol (28% loading) and DMSO (35% loading) as solvents revealed the amorphous nature of these samples. A rutin peak was detectable in the physical mixture with 3% rutin. The crystalline rutin peaks were only detectable for SP53D loaded with 28% rutin using ethanol as a solvent, which is in agreement with the DSC data. Even the silica with a high loading of up to 35% (loaded with DMSO) was a stable, amorphous material. As mentioned, the industrial feasibility is more important maximizing the achievable loading. To be on the safe side, 20% loadings produced with ethanol were used for determination of the saturation solubility, and this is recommended as the maximum loading for commercial use.

**Figure 5:**
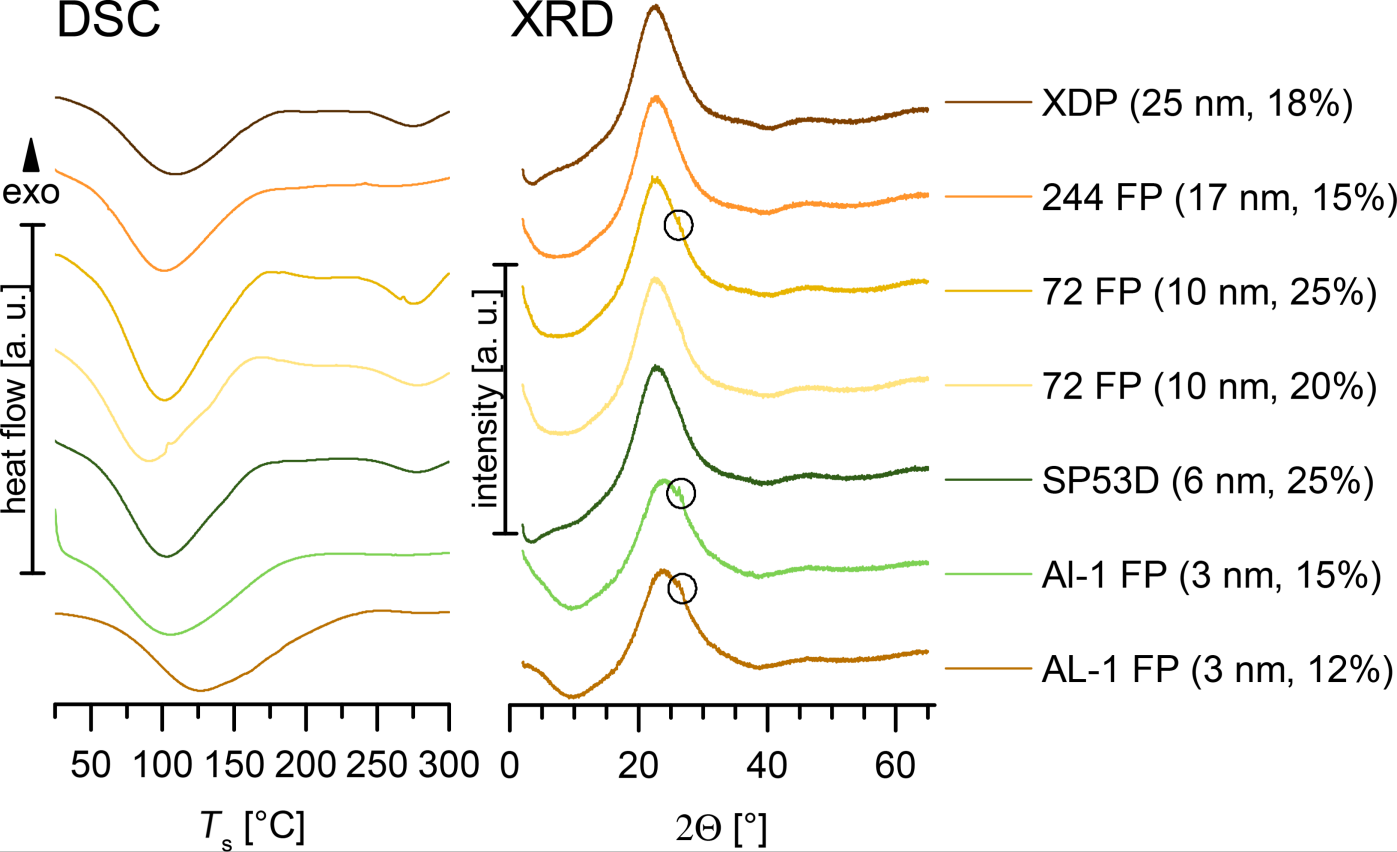
DSC thermograms (left) and XRD diffractograms (right) for selected silica nanoparticles, all loaded using ethanol as the solvent for rutin, showing curves with highest amorphous loading. For 72 FP (10 nm pores) the curves obtained with the overloaded system (25%) are also shown, and for AL-1 FP, only overloaded curves are presented (pore size 3 nm, 12% and 15% loading).

Figure 5 shows a comparison between all investigated silica samples, where the DSC curves (left) and XRD patterns (right) are plotted with the highest achievable loading of amorphous material for each silica type, showing no peaks for appropriately loaded silica samples. Only for 72 FP (the curve with no peaks (20% loading), and the curve with peaks are plotted (system overloaded, 25%). For AL-1 FP samples (the silica with the smallest pore size of 3 nm) only the overloaded curves are shown (12% and 15%). The comparison between DSC and XRD reveals that XRD was more sensitive to detecting crystallinity. In all overloaded systems, where peaks could be detected by XRD, no peaks could be seen in DSC. Thus, applying both methods in parallel was necessary. In conclusion, silica with a very small pore size of 3 nm are deemed to be less suitable. The loading of the other silica materials was generally between 15% and 25%.

### Light microscopy and scanning electron microcopy

**Figure 6:**
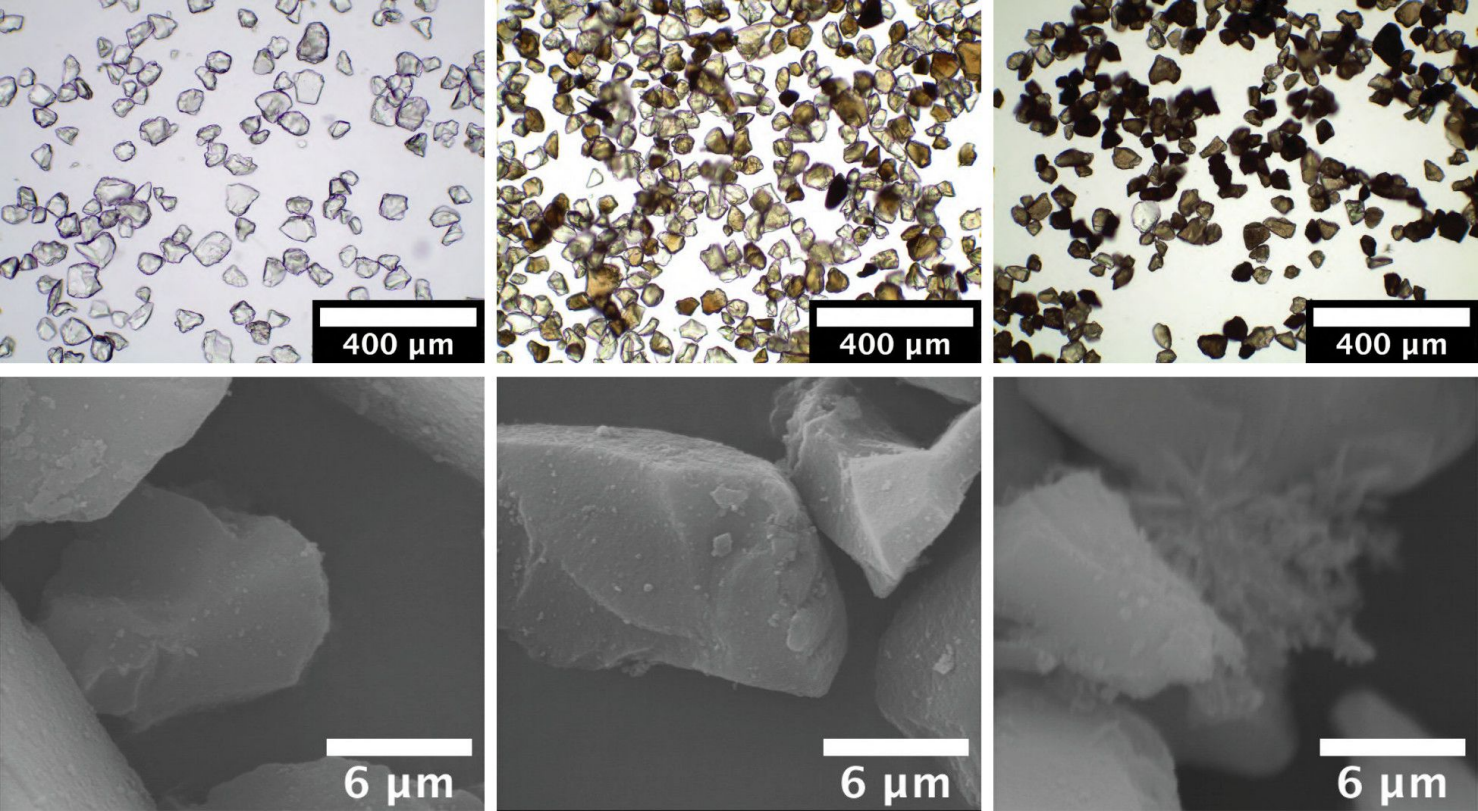
Upper row: light microscopy pictures of silica XDP 3050 unloaded (left), loaded with the maximum amorphous fraction of 18% (middle) and overloaded silica (20% rutin, right). Lower row: corresponding SEM pictures (from left to right).

Light microscopy is readily accessible and fast to perform; thus, it was also used to follow the loading process. Light microscopy provides a general overview with one glance. When rutin crystals are present outside of the silica particles, they should be easily detectable. Also, it was expected to see changes on the surface of the silica, i.e., evidence of deposition of rutin. Figure 6 (upper row) shows exemplarily images of XDP 3050 particles. When unloaded, the silica particles appear translucent (upper left); loading rutin into the pores results in the coloring of the particles (upper middle). It is apparent that after loading some particles are still relatively translucent, while other particles become light to medium colored, although very dark colored particles can also be seen. That means that not all particles are loaded to the same degree, and thus there is a wide loading distribution among the particles. This is important for understanding the process. When adding more rutin solution, the heavily loaded particles will first show a crystalline fraction (due to rutin crystallizing on the surface). The overloaded system with 20% rutin (upper right) shows a majority of dark colored particles.

In Figure 6 (lower low), the SEM of unloaded silica (lower left) shows a smooth surface, almost free of fine particulate material. The maximum loaded particles (lower row, middle) show some fine particulate material on the surface. From light microscopy it can be concluded that there is a distribution in the degree of particle loading. Thus, the problem in SEM analysis is to distinguish to which particles the microscope is focusing on. In the sample XDP 3050 with overloaded silica (20% loading, lower right), the particles can be found with pronounced fine particulate material on the surface of some of the 50 µm silica particles.

In conclusion, light microscopy is a suitable complementary tool for evaluating the loading process, while SEM provides additional insight into the mechanism behind the loading process, but is not essential for batch monitoring during an industrial production process.

**Figure 7:**
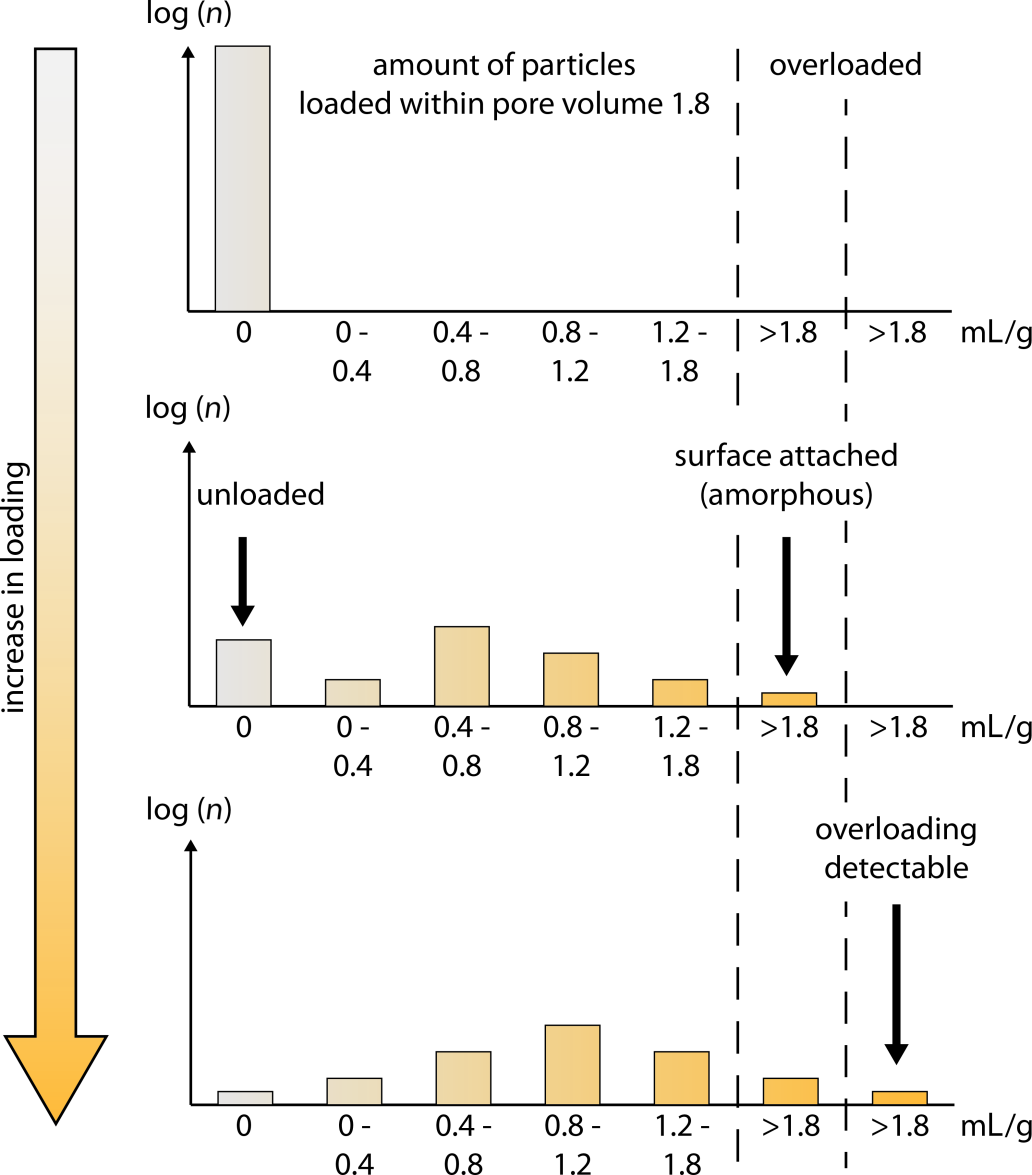
Description of the silica particle loading mechanism showing the distribution of differently filled particles as a function of loading. Upper plot: in the unloaded state, all particles fall within the 0 mL/g fraction. Middle plot: maximum loading condition where no crystallinity was detectable by XRD; the particles are filled to various extents, possibly with some being overloaded (>1.8 mL/g). Lower plot: some particles are obviously overloaded; the population contains a particle fraction in which detectable crystallization occurred.

Based on this analysis, a loading mechanism is proposed (Figure 7) using the example of a silica material with a pore volume of 1.8 mL/g. At the beginning (before loading), all pores are empty (0 mL/g, see the upper graph in Figure 7). As seen from the light microscopy pictures (after the loading process), obviously some of the particles remain unfilled (light/transparent particles), some are partially filled, and some are heavily filled (very dark particles). This is represented in Figure 7 in the middle graph. The degree of solvent penetration into different particles varies, leading to particles remaining unfilled (at 0 mL/g filling) or particles that are partially or medium filled (0–0.4 and 0.4–0.8 mL/g, respectively). There are also particles with higher pore volume filling (0.8–1.2 and 1.2–1.8 mL/g) and also particles with completely filled pores (filled volume 1.8 mL), which additionally have rutin on the surface. This implies that the rutin volume per gram of particles for these samples is >1.8 mL/g (Figure 7, middle). This surface material is amorphous, since no evidence of crystallinity was detected.

If too much rutin solution is used and evaporation continues, rutin continues to precipitate on the surface of completely loaded particles. When rutin deposits as a thin layer on the surface, the rutin stays amorphous; when the layer thickness increases above a critical threshold, crystallization occurs which can be detected by DSC/XRD (Figure 7, lower plot). For the use of the particles on the skin one could even argue that overloaded systems can be used, because the vast majority of the particles contain the amorphous phase active agent.

### Loading efficiency – recovery rate

**Figure 8:**
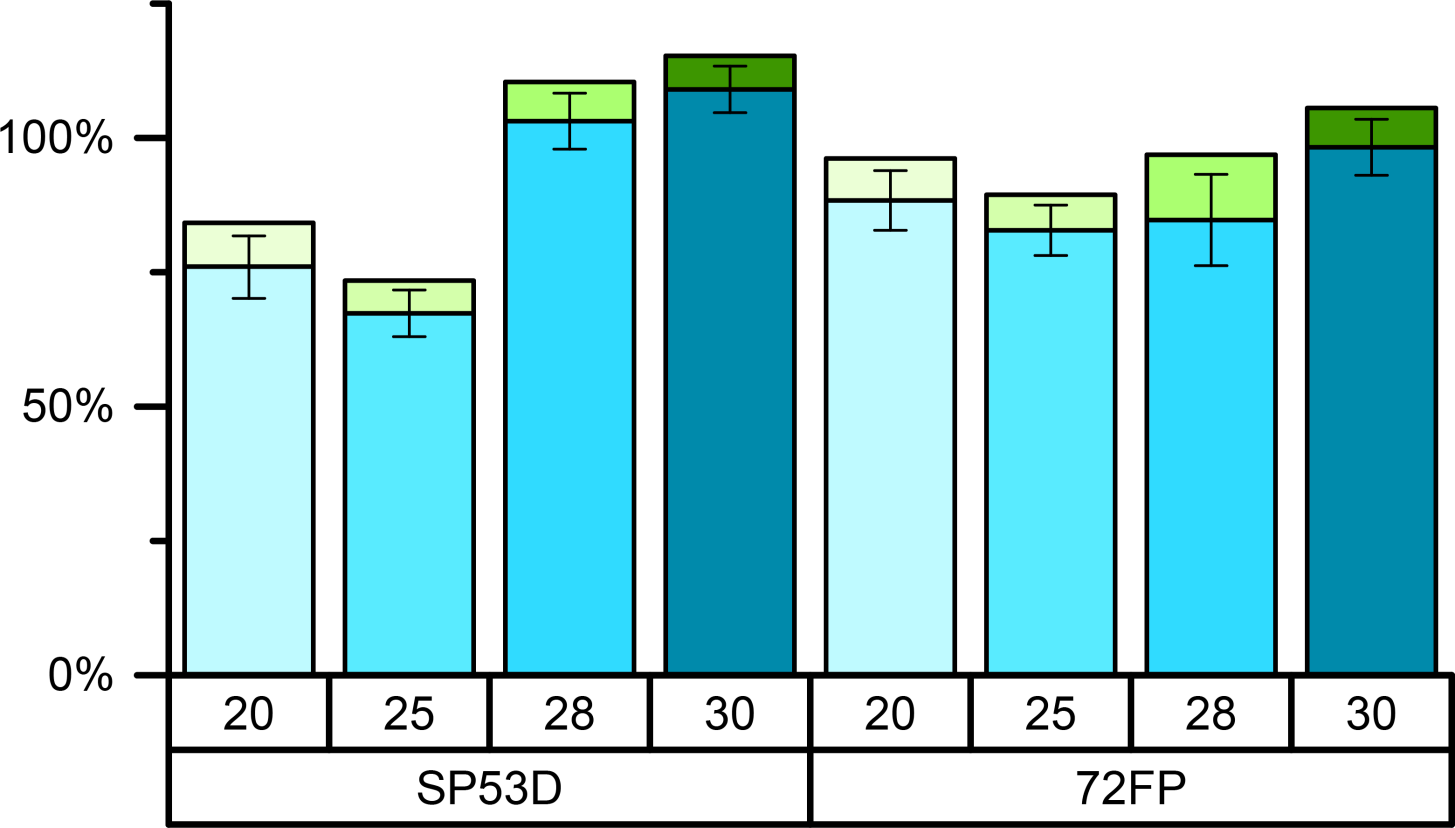
Recovered fraction of rutin after extraction from silica SP53D (left) and 72 FP (right) with rutin loadings of 20%, 25%, 28% and 30%, as measured with HPLC (blue) and spectrophotometry (green). The standard deviation is shown as the mean of all measurements (HPLC *n* = 2, UV *n* = 1).

It was also investigated whether the initial content of rutin in the different particle types could be recovered. The rutin was extracted with a solvent, and the amount was determined by spectrophotometry and HPLC. The coefficient of determination was 0.9943 for HPLC and 0.9921 for UV spectrophotometric measurements. Figure 8 shows the amount recovered for two silica samples, SP53D and 72 FP, with loadings from 20% to 30%. The SP53D and 72 FP silica are shown because of their high loading (>20%) and suitability for amorphous rutin stabilization.

At lower loadings (i.e., 20%, 25%) a minor fraction could not be recovered. It is reported in the literature that, due to the large surface area, a delayed release takes place, where some active agent is quite firmly bound to the surface and is thus not released [34]. Thus, the absolute retrieved amount increases with increasing loading. Additionally, the higher recovery rates for samples with a certain crystallinity is remarkable. This could explain the higher recovery rate at higher loadings of 28–30%. The silica sample with the smaller pores (3 nm, AL-1 FP) showed at low maximum loading (12%) and a recovery rate of only about 35%. The recovery rate was also analyzed by HPLC, and the recovery fractions are all slightly below, but in agreement with, the values obtained using spectrophotometry, as shown in Figure 8.

The difference in the results obtained from UV spectrophotometry and HPLC analysis can be explained by the extraction times applied for HPLC and UV spectrophotometry samples. In general, a higher recovery rate was found in the spectrophotometry analysis due to the longer extraction time (about 30 min for HPLC analysis versus 2 h for spectrophotometry). The incomplete release of the material from the small pores and the influence of extraction time is relevant for the in vivo situation. Dermal formulations are often applied for 10–12 h (e.g., morning application to the face, followed by face washing in the evening). To have a release as complete as possible, larger pores are thus favorable for dermal products. Apart from the consequence for dermal delivery, the data show that for better reproducibility, the extraction procedures need to be exactly identical.

### Solubility determination of rutin dissolved from smartPearls

**Figure 9:**
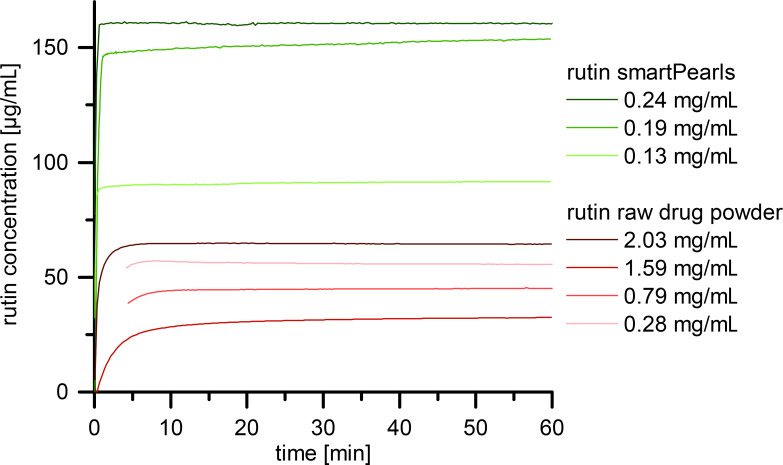
In situ determined saturation solubility of rutin raw active agent powder (lower curves, red) and amorphous rutin loaded into silica as smartPearls (upper curves, green) with various rutin concentrations.

The saturation solubility, as reported by Mauludin et al., for rutin nanocrystals and rutin raw material is about 130 µg/mL [35]. Thus, the solubility of the raw active agent powder is attributed to the presence of nanometer-sized rutin particles in the raw material. Measuring the solubility of the rutin raw powder with the setup used in this study at 25 °C yielded a saturation solubility from 30 to 70 µg/mL at pH 5.5 ± 0.5 (Figure 9, lower curves). Apart from the nanometer-sized material, the general high values reported in the literature can be attributed to dissolution in a higher pH (6.8) and at a higher temperature (37 °C) than that used in this study (pH 5.5 for skin products and 25 °C). Additionally, the in situ measurement with baseline correction and the 0.15 molar NaCl solution used in this study also led to differences from the values reported in the literature. The assumption that nanometer-sized rutin particles are present in the raw material is supported by the fact that the measured *C*_s_ value increased from 30 to 70 µg/mL with increasing amount of rutin powder (from 0.28 mg/mL to 2.03 mg/mL) added to the solvent (i.e., more nanometer-sized rutin was added which dissolved and led to this increase in the measured solubility).

In contrast to the rutin powder, in this study, an increased *C*_s_ of about 160 µg/mL was obtained with amorphous rutin dissolved from smartPearls. This implies an increase by about a factor of two higher than the respective raw active agent powder. This higher *C*_s_ value leads to an increased concentration gradient between the dermal formulation and skin and thus to an increased flux of rutin into the skin.

The dissolution kinetics of rutin from the smartPearls is much faster than from the raw rutin powder. With smartPearls, the solubility saturation is reached after almost 1 min, for the raw rutin powder, it takes 5–10 min. For the dermal formulation the kinetics is not relevant, only the saturation solubility, *C*_s_. The smartPearls are added in the preparation of the dermal formulation, and a saturated state will occur in the formulation. When applied to the skin, it can be predicted that the rate limiting step is the slow diffusion of dissolved rutin into the skin. There will be no difference if the diffused rutin molecules are replaced in the dermal formulation by faster or slower dissolution.

In vitro skin penetration studies showed an even higher increase in penetration when a gel with smartPearls was compared to a gel with rutin powder (pig skin penetration test, tape stripping [24]). Especially in the deeper skin layers, an increase by a factor of about four was observed.

### Concentration of smartPearls in final market products

When adding smartPearls to the water phase of a dermal product, some portion of the rutin in the pores will dissolve, forming a supersaturated solution. Some portion must remain undissolved in the pores in order to provide a rutin source. When rutin dissolved in the water phase has penetrated into the skin, it should be replaced by new rutin molecules dissolving from the pores. With this scheme, a constant supersaturated state will be maintained. Based on this, the rutin concentration required for a dermal product can be calculated.

The measured saturation solubility of rutin from smartPearls is 150 µg/mL (i.e., 0.15 mg/mL). This will be the amount of rutin that dissolves when adding the smartPearls to the formulation. In addition, one needs a rutin loading in the pores. Thus, it is recommended to use about two times the amount dissolved. For highly penetrating active agents, the required amount stored in the pores might even be higher. Based on this, a minimum of about 0.5 mg/mL in the final product is required. Assuming a low loading of the smartPearls of 10% (w/w), a total of 5 mg of loaded smartPearls need to be added per mL (g) of product (i.e., 5 g/1 kg product).

### Definition of large-scale production parameters

Based on this, producing a 100 kg batch requires 500 g of smartPearls; assuming only 10% loading, this corresponds to 50 g of rutin powder. In the production process, this 50 g of rutin has to be dissolved in ethanol. Based on an ethanol solubility of 2%, 2.5 kg of ethanol are required. In this rutin solution, 450 g of unloaded silica particles are dispersed. For evaporation, a rotary evaporator with about 2.5 L solvent evaporation capacity is required, i.e., a rotary evaporator with a volume of about 20 L.

Large rotary evaporators are available up to volumes of 200 L (e.g., from GlasKeller AG, Basel, Switzerland). That means that in one loading process, 5 kg of smartPearls are sufficient to produce 1000 kg of final product. This proposed production scheme indicates that the industrial production of smartPearls is feasible.

## Conclusion

For monitoring and understanding the mechanism behind the loading process, a combination of DSC and XRD techniques is most suitable. In this study, XRD was found to be more sensitive to understanding the material crystallinity than DSC.

The particles demonstrated a maximum loading of amorphous-phase rutin of 25% when loaded using ethanol as the solvent. With DMSO as the solvent, a loading of up to 35% was possibly. However, DMSO is difficult to remove because of its high boiling point, and finally, ethanol is preferable because of its better skin tolerability, price and ease of processing.

To ensure complete amorphization a loading of 20% rutin is recommended given that recrystallization of excess rutin was first detected at loadings ≥28%. The saturation solubility of the loaded material was found to increase by a factor of two with just one tenth the amount of amorphous rutin compared to raw active agent powder.

Light microscopy contributed to the understanding of the loading process. It efficiently revealed that the pore filling follows a distribution in the degree of filling (ranging from unloaded to highly loaded). Only a portion of the particles achieve complete loading, and these are the particles that tend to show the first crystalline fractions.

Regarding the ease of processability, as predicted, the smaller particles (such as AL-1 FP, 7 µm diameter) have a higher tendency to agglomerate, and in combination with their small pore size of 3 nm, the maximum loading is low (<12%). Better processability was observed for larger particles with larger pores and pore volume, where the best results among the silica materials studied was found with Syloid^®^ XDP 3050.

Due to the solubility properties, the amount of 5 kg loaded smartPearls required for one ton of final product is relatively low. However, with an established process, large-scale production should be possible using commercially available industrial-sized 50 L evaporators (i.e., the process is scalable). To reproduce the small-scale laboratory results on a large scale, only the temperature and vacuum parameters need to be set so that the same amount of solvent evaporates in a given time (i.e., the Ostwald–Miers range is satisfied).
